# 

**Published:** 1895-03-09

**Authors:** 


### CONTENTS.

**A Deadly Disease of Our Own Times** - - - - - 395

**Wound Infection** - - - - - - - - - - - 396

**Modern Medico-Psychology and Psychiatry.**—By T. S. CLOUSTON, M.D.—States of Fixed Delusion - - - - 397

**The Panacea in English Medicine.** III.—Tar - - - 398

**Medical Progress and Hospital Clinics:**

Club-foot: Its Nature and Treatment - - - - - - 401

PROGRESS IN MEDICINE.—Infective Diseases - - - - 403

PROGRESS IN SURGERY.—Hernia. II. - - - - - - 404

PROGRESS IN DERMATOLOGY - - - - - - - - 405

NEW APPLIANCES AND THINGS MEDICAL - - - - - 406

**Notes and News** - - - - - - - - - - - 400

**Annotations.** Influenza Convalescents—Football Players' Impetigo (?)—The Factories and Workshops Bill - - - - 399

**The Institutional Workshop:**

HOSPITAL CONSTRUCTION.—New Eye Infirmary, Sunderland - 407

HOSPITAL ADMINISTRATION —The Derbyshire Royal Infirmary - 408

PRACTICAL DEPARTMENTS.—Heating and Ventilation - - - 408

HOSPITAL FESTIVALS AND MEETINGS - - - - - - 409

**Scraps and Gleanings** - - - - - - - - - - 410

**Editor's Letter [illegible]ox** - - - - - - - - - - 411

**The Book World of Medicine and Science** - - - - 411

### EXTRA SUPPLEMENT.—THE NURSING MIRROR.

**News from the Nursing World.**—A Queen in Isolation—Women Lecturers—Probationers' Examination at Lambeth—Maternity Work at Clapham—Life's Failures—Nurses at York—Queen's Nurses at Southampton—Cambridge—Night Nursing Needed—Nursing in Wales—District Nursing in Ireland—Nursing at Bishop Auckland—St. Patrick's Nurses' Home—Faithful to Duty—A New York Club—Short Items - - - - - clxvi

**Burdett's Official Nursing Directory** - - - - clxvii

**Where to Go** - - - - - - - - - - - - - - clxvii

**Elementary Anatomy and Surgery for Nurses.**—By W. MCADAM ECCLES, M.B., M.S., F.R.C.S. VIII.—Fractures in General - - - - - - - - - - - - clxvii

**Wants and Workers** - - - - - - - - - - - clxvii

**Notes from St Helena** - - - - - - - - - clxviii

**Nursing in Cairo** - - - - - - - - - - - - clxix

**Everybody's Opinion** - - - - - - - - - - clxix

**The Book World for Women and Nurses** - - - - clxx

**For Reading to the Sick** - - - - - - - - - clxx

